# Long term follow-up of a full- arch rehabilitation with an implant-supported overdenture on four zirconia implants and a zirconia bar. A case report

**DOI:** 10.4317/jced.59362

**Published:** 2022-04-01

**Authors:** Jesús Peláez, Judith Gelfo-Flores, Maria-Isabel Albanchez-González, Santiago Bazal-Bonelli, Carlos López-Suárez, Rubén Comino-Garayoa, Ramiro Mallagray-Martínez, Jorge Cortes-Bretón Brinkmann

**Affiliations:** 1 Adjunct Professor. Department of Conservative Dentistry and Orofacial Prosthodontics, Faculty of dentistry, Complutense University of Madrid, Spain; 2 Private practice Madrid, Spain; 3 Master Program Buccofacial Prostheses and Occlusion, Faculty of Dentistry, Complutense University of Madrid, Spain; 4 Master Program Oral Surgery and Implantology, Faculty of Dentistry, Complutense University of Madrid, Spain; 5 Associate Professor. Department of Conservative Dentistry and Orofacial Prosthodontics, Faculty of dentistry, Complutense University of Madrid, Spain; 6 Master Program Buccofacial Prostheses and Occlusion, Faculty of Dentistry, Complutense University of Madrid, Spain; 7 Private practice Madrid, Spain. Gómez Ulla Defence Hospital. Oral and maxillofacial surgery service; 8 Associate Professor. Department of dental Clinical Specialties, Faculty of dentistry, Complutense University of Madrid, Spain

## Abstract

This clinical report describes a mandibular full-arch rehabilitation by means of an implant-supported overdenture on four zirconia implants. A female patient with an edentulous mandibular arch attended our dental clinic seeking a metal-free mandibular restoration. After oral and radiographic evaluation, four one-piece zirconia implants with conical abutments were placed in the intermentonian region. After a 3-month osseointegration period, an acrylic overdenture with plastic matrices was manufactured, supported by a zirconia bar cemented to the conical abutments of the zirconia implants. Radiographic and clinical follow-up after 12 years observed adequate implant evolution, without any signs of peri-implant disease. Maintenance events were principally the periodic replacement of the plastic matrices and prophylaxis.

** Key words:**Zirconia implants, overdenture, full-arch rehabilitation, dental implants, case report.

## Introduction

Although titanium implants demonstrate excellent biocompatibility and numerous possibilities for restoring partially and completely edentulous patients, several questions have arisen recently regarding some problems that occur with this type of implant, pointing to a need for an alternative (1). The first issue is hypersensitivity to titanium. Several studies have indicated that, although uncommon, some patients can develop clinical signs of allergy to titanium (1,2). The prevalence of titanium hypersensitivity has been estimated at only 0.6% (3), but it does present a real possibility and will provoke a series of symptoms (3). The mechanism that can trigger an allergy to titanium implants is the corrosion process that has been shown to occur in the presence of saliva and bacterial biofilms (4). The ions derived from this process can form complexes with native proteins and act as allergens when they come into contact with mucosa or skin (5).

Another drawback of titanium implants is its grey color, especially when placed in anterior areas with a thin gingival biotype (1,6). Nowadays, higher esthetic demands and apprehension toward titanium hypersensitivity have led to a growing demand for metal-free restorations. Ceramic materials have been proposed as potential alternatives (1).

Currently, tetragonal zirconia polycrystal, particularly yttrium oxide (yttria) stabilized zirconia, is the ceramic of choice for ceramic dental implants (1,7). The white, opaque color as well as reports of good biocompatibility, low bacterial plaque accumulation, little inflammatory infiltrate, and good soft tissue integration, make it a material of great interest for use in implantology. Zirconia also presents several favorable physical properties such as low thermal conductivity, high flexural strength (900–1,200 MPa), favorable fracture resistance, and resistance to wear and corrosion. However, early failure rates of zirconia implants seem to be higher than with titanium implants, and studies reporting long-term outcomes are scarce (1). Clinical research involving novel prosthodontic designs supported by zirconia implants, particularly in completely edentulous patients, is also limited (8).

The present case report describes the use of four zirconia implants to support an overdenture and its evolution over a 12-year follow-up. As far as the authors are aware, this is the first ever case report to describe the use of four zirconia implants supporting this prosthodontic design with a long follow-up period.

## Case Report

A 71-year-old female patient, without relevant medical antecedents, visited our dental clinic in June 2008 seeking treatment to improve her oral health status. The only remaining teeth were the upper incisors and lower canines (#7, #8, #9, #10, 22# and #27). She refused any treatment options involving titanium implants and metal retainers. Clinical and radiographic examination observed advanced bone loss around teeth #7, #10, #22 and #27, as well as advanced resorption of the residual alveolar ridge in the maxillary arch and in the posterior regions of the mandibular arch, which did not allow implant placement. In the maxillary arch, it was decided to extract #7 and #10 and to fabricate a removable partial denture. In the mandible, since the patient declined metal materials and mucosal support was insufficient, it was decided to place an implant-supported prothesis on four zirconia implants immediately inserted in the intermentonian region following the extraction of #22 and #27. Due to antecedents of poor oral hygiene and the presence of an atrophied mandible an overdenture was preferred to a fixed restoration. Furthermore, the patient refused to undergo more exhaustive regenerative surgical procedures. The patient accepted that this prosthetic design was new and its use in cases such as hers had not been described much in the literature. She also understood and accepted the importance of attending periodic follow-ups.

1. Treatment planning

**Figure 1:**
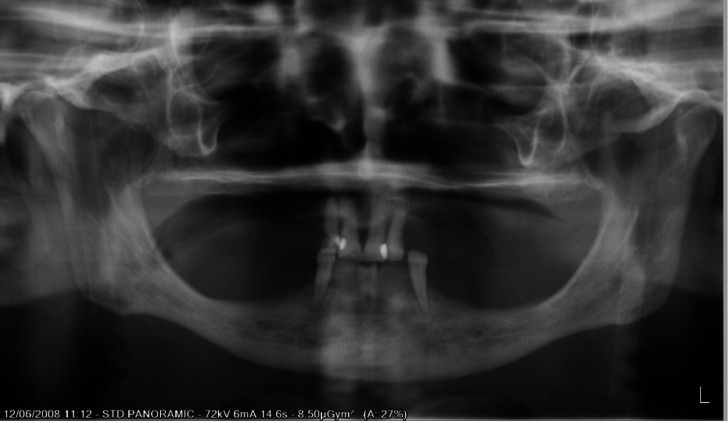
Orthopantomography of the patient on his first visit.

An orthopantomography (Fig. 1) and Cone Beam Computed Tomography were taken to assess the feasibility of implant placement. It was decided that four zirconia implants could be safely inserted in the intermentonian region to support the implant-supported overdenture. The patient was given full information about the procedure and gave consent to undergo treatment in awareness of the possible risks and complications of this novel prosthetic design.

Prior to the surgical phase, all the information needed to fix patient dental casts in a semi-adjusTable articulator was recorded: impressions with irreversible hydrocolloid (Hydrogum 5, Zhermack), facebow and centric relation wax bite registration. Record bases with occlusion rims were manufactured, which allowed the registration of the patient’s occlusal vertical dimension and centric relation. Once the casts were fixed in the articulator, a wax try-in of the future acrylic teeth was realized. This step served to check that prosthetic space was sufficient for the overdenture and the conical abutments of the zirconia implants, and that these abutments would not interfere in the positions of the teeth. The wax try-in was duplicated to fabricate a surgical guide and the complete denture itself.

2.Surgical procedures

**Figure 2:**
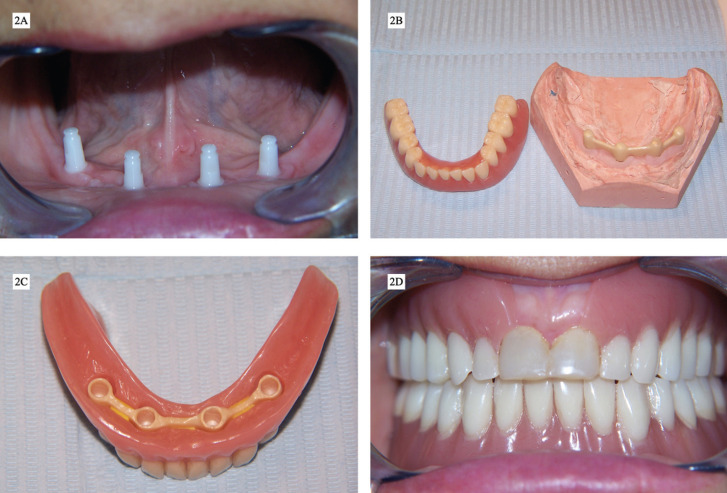
A. Zirconia implants after placement and soft tissue healing, B. Zirconia bar and acrylic overdenture before the delivering, C. Intaglio surface of the overdenture with the plastic matrices and the zirconia bar, D. Upper and lower prosthesis just after the delivering.

Four one-piece zirconia implants with conical abutments (Bredent White Sky), two of 3.5x10 mm at positions #23 and #26, and two of 3.5x12 mm at positions #21 and #28, were placed in the intermentonian region in a single surgical session by an experienced oral and maxillofacial surgeon, immediately after the extraction of #22 and #27 (Fig. 2A). A mid-crestal flap was raised for implant placement at the positions #21, #23, #26 and #28. The surgical site was sutured with 5/0 polyamide multifilament suture (Supramid, Aragó, Barcelona, Spain), removed 10 days after surgery.

3. Prosthodontic procedures

Immediately after surgery, the intaglio surface of the immediate complete denture was relieved (holes were made at the positions of the zirconia implant conical abutments) and relined with a tissue conditioner (Ufi-gel SC, Voco). After three months’ osseointegration (conventional loading protocol), lower arch impressions were made with polyether (Impregum Penta, 3M ESPE), and with irreversible hydrocolloid (Hydrogum 5, Zermack) for the upper arch. At the next appointment, all the records needed to mount the working casts in the articulator were taken with the help of a previously manufactured lower record base with occlusal rim: occlusal vertical dimension, centric relation, and facebow. Before the fabrication of the zirconia bar, a wax try-in with acrylic teeth was made in the laboratory to check occlusion and functional and esthetic parameters. Once this wax try-in was approved, a zirconia bar, splinted to all the conical abutments of the zirconia implants, was fabricated. This zirconia bar was designed with Hader cross-section geometry (9) to avoid rotation movements around it, as this prosthesis would not be supported by mucosa. The zirconia bar’s marginal and passive fit were checked clinically and radiographically. Lastly, a mouth test of the zirconia bar along with the wax try-in was realized before the acrylization of the overdenture and the incorporation of the attachment systems, which consisted of three plastic matrices adapted to the rounded surface of the three segments of the zirconia bar (Fig. 2B,C). On the day of prosthesis delivery (Fig. 2D), the zirconia bar was cemented to the conical abutments with self-adhesive resin cement (RelyX Unicem Automix, 3M ESPE) and occlusion was checked with 12 and 8 µm articulating paper. The occlusal pattern selected was conventional bilaterally balanced occlusion, since the maxillary prosthesis was completely supported by mucosa.10 Instructions for use and hygiene were given to the patient.

4.Recalls and follow-up

**Figure 3:**
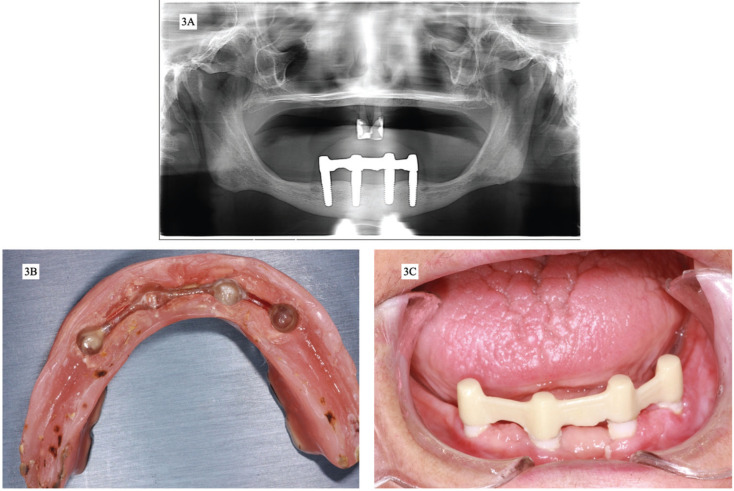
A. Orthopantomography after 12 years of implants placement, B. Overdenture after 12 years of use and before plastic matrices replacement and prophylaxis, C. Zirconia implants and bar after 12 years of placement and before routine prophylaxis.

Rigorous follow-up of the patient continued throughout the healing period, and subsequent follow-up visits were scheduled. The patient was recalled for check-ups at 15 days, 1 month, and thereafter every 6 months. After 12-years follow-up, no peri-implant bone loss has been observed in radiographic examination (Fig. 3A). Maintenance events have consisted mainly of the periodic replacement of the plastic matrices, whenever significant wear has been detected, and the cleaning and polishing of the acrylic prosthesis, bar and zirconia implants whenever calculus accumulation have been observed (Fig. 3B,C). The patient has expressed her satisfaction with the treatment both in terms of function and esthetics.

## Discussion

Little clinical research has reported the clinical behavior of novel prosthodontic designs for overdentures supported by zirconia implants. Nevertheless, they constitute a potentially viable treatment option for edentulous patients sensitive to titanium implants and metallic attachment systems, or those who refuse metallic restorations (8).

Conventional implant-supported overdenture designs consist of two splinted or non-splinted interforaminal mandibular implants and four maxillary implants. Placement of additional posterior implants, as in the present case, alters this common type of implant-and-mucosa supported overdenture, making it a fully implant-supported overdenture with correspondingly different biomechanical behavior (10). In a clinical trial carried out by Osman *et al*. (11) with 24 patients, implant-supported overdentures on one-piece zirconia implants with ball abutments were compared with the same design of overdenture on titanium implants. Each participant received three mandibular implants in a tripodal design (mid-line and both molar areas) and four maxillary implants in a quadrilateral design (mid-line, mid-palatal and both premolar regions). After a 1-year follow-up, no significant difference in survival rates was found, although there were more implant fractures and significantly more marginal bone loss in the zirconia group. Nevertheless, in both groups the overall survival rates were too low (81.3% in the titanium group and 71.3% in the zirconia group) to recommend the design for routine clinical practice. The authors could only recommend one-piece zirconia implants in cases of proven allergy to titanium or for patients who specifically request metal-free rehabilitations. Maintenance events were principally, as in the present case report, the replacement of the plastic matrices.

Brandt *et al*. (12) conducted a retrospective study of 126 telescopic overdentures supported on four zirconia conical abutments with an observation period of 11.5 years. Although their results are not totally relevants to the present case report as the overdentures design was different, 5-year prosthetic survival was 96.9%.

From the biomechanical point of view, an *in vitro* study by Osman *et al*. (13) used finite element analysis to compare one-piece zirconia and titanium implants supporting maxillary overdentures on four implants. The stress and strain values in peri-implant bone for both types of implants were comparable, and so it was concluded that zirconia implants may be a potential alternative for supporting overdentures.

Regarding the limitations of this technique, it should be noted that one-piece zirconia implants require sufficient prosthetic space to accommodate the abutments. In addition, implant placement may not meet the prosthodontic requirements (1) and secondary correction of the shape or size of the abutments by grinding is not recommended as this severely decreases zirconia’s fracture resistance (1,14). The screw-retained two-piece zirconia implants tested to date have shown higher failure rates and lower fracture resistance than titanium systems, so most zirconia implants systems are one-piece. Another limitation of these implants is that cementation is the only option for connecting prosthodontic elements to the implants, increasing the risk of excess of luting cement remaining in the periodontal area, and making it harder to retrieve these prosthodontic elements in case of failure. Moreover, a clinical study found that one-piece zirconia implants of narrow diameter (3.25mm) present a higher risk of fracture (12,15). This adds bone availability to the list of factors that must be taken into consideration when it comes to choosing the option of zirconia implants. In light of these limitations, proper case selection and planning, as well as adequate handling of the material, would appear critical to a successful outcome.

## Conclusions

The present case report describes mandibular full-arch rehabilitation by means of an implant-supported overdenture on four one-piece zirconia implants with conical abutments, with a zirconia bar cemented to them. Within the limitations of this clinical case report, the technique was found to be a successful alternative to traditional overdentures on titanium implants. Radiographic and clinical follow-up over 12 years observed adequate implant evolution, without signs of peri-implant disease; maintenance events were principally the periodic replacement of the plastic matrices. The literature lacks information and evidence regarding this technique. Nevertheless, the present case has shown that satisfactory results can be achieved over a 12-year follow-up, providing case selection protocols and treatment planning are adequate.
